# Public opinion towards castration without anaesthesia and lack of access to pasture in beef cattle production

**DOI:** 10.1371/journal.pone.0190671

**Published:** 2018-01-05

**Authors:** Dayane Lemos Teixeira, Rafael Larraín, Oscar Melo, María José Hötzel

**Affiliations:** 1 Departamento de Ciencias Animales, Facultad de Agronomía e Ingeniería Forestal, Pontificia Universidad Católica de Chile, Santiago, Chile; 2 Laboratório de Etologia Aplicada, Departamento de Zootecnia e Desenvolvimento Rural, Universidade Federal de Santa Catarina, Florianópolis, Brazil; 3 Departamento de Economía Agraria, Facultad de Agronomía e Ingeniería Forestal, Pontificia Universidad Católica de Chile, Santiago, Chile; Universidade do Porto Instituto de Biologia Molecular e Celular, PORTUGAL

## Abstract

Recent publications have shown that citizens in developing nations are gaining interest in farm animal welfare. The aims of this study were to assess the opinion of Chilean citizens about surgical castration without anaesthesia and lack of access to pasture in beef cattle production, to investigate how involvement in livestock production influences opinions, and to evaluate if different types of information would affect their opinion towards these management practices. The study was carried out in the Metropolitan Region of Santiago, Chile, and consisted of two surveys with 400 participants in each study. The first one used an online, self-administered questionnaire and the second one used a face to face questionnaire. The second questionnaire had four information treatments assigned randomly to survey participants (no information; negative information; negative and positive information; positive information). Most participants were aware that the two management practices are common in beef production systems and were opposed to them. Involvement in animal production was associated with greater acceptance of both management practices and participants that had visited a beef production farm before the study were more likely to support castration without anaesthesia in Survey 1. Belonging to any socioeconomic group and providing negative or positive information had no impact on participants’ opinion. The results show a disconnection between the views of participants recruited for this study and beef production systems that do not provide pain control for male cattle surgical castration or provide little or no access to pasture.

## Introduction

In the last decades, the growth of the livestock sector has been accompanied by intensification of agriculture, and the fast adoption of technologies and of confinement and caged housing [1]. However, public rejection of some aspects of intensive animal production systems has led to the development of regulation and industry actions associated with animal care at the farm level [2]. In developed countries, the general public has become increasingly interested in farm animal welfare [3, 4]. Although some recent publications have shown that citizens in developing nations are gaining interest in this aspect of food animal production [5–8], public interest in the topic is considered an emerging issue and further investigation is urged due to rapidly increasing demand for livestock products [2].

Surgical castration, which involves the removal of the testes, is performed to improve product quality and management of male beef calves; the procedure has been proven to cause significant pain and stress [9, 10]. Nonetheless, most producers still routinely surgically castrate cattle without anaesthesia in Chile [11]. Previous studies investigated consumer opinion towards surgical castration of male pigs and alternative methods in Europe [12–15]. However, public and consumer attitude towards castration without anaesthesia in beef cattle has not been investigated.

Chilean consumers have a positive perception towards meat from pasture-fed animals [6], and acceptability ratings are higher for beef with low marbling levels and beef from grazing animals than beef with high marbling levels and feedlot systems, respectively [16]. Purchasing decisions by Chilean beef consumers are highly influenced by quality assurance, but meat produced under protocols that consider animal welfare is also highly attractive for this population [17]. On the other hand, although most of Chilean cattle are finished in grazing systems [18], a significant proportion of Chilean beef cattle from the central areas of the country goes through feedlots, specially due to the short grass-growing season for finishing and that because grain and agricultural by-products are less expensive. As fruits and crops production continue to expand into once traditional cattle areas, it is possible to expect an intensification of the beef production systems due to an increased animal protein demand [1]. In other countries, a number of studies indicate public opposition to systems that do not offer access to pasture for dairy cattle [5, 19, 20]; however, in South America no study has investigated public opinion regarding beef cattle rearing systems.

Previous studies suggested that public rejection of some production practices is associated with low awareness and lack of knowledge of how food producing animals are reared [21]. However, providing information regarding layer hens’ housing [22], gestation stall housing for sows [23], and surgical castration of piglets [13] did not improve overall lay citizens’ attitudes. In fact, when lay citizens where provided with information justifying the practices and discussing limitations of zero-grazing systems in dairy cattle, they showed higher rejection of this practice [5]. The fact is that information both in support of or rejecting a particular practice may affect public opinion due to citizens’ ignorance of the current practices of animal production, by making them aware of the existence of a practice they did not know and they find surprisingly inappropriate [24].

Stakeholders associated with livestock often support some practices considered contentious by lay citizens, as for example castration [25–27], tail docking [28] and calf dehorning [29] without pain control, early cow calf separation [30], or housing sows in gestation stalls [28]. Common arguments offered by these stakeholders are that these practices are important or essential to achieve production and economic goals (e.g., [25, 28, 29], and that lay citizens’ ignorance of farming realities is the reason why they reject them [31, 32].

The main aim of this study was to assess the opinion of Chilean citizens about two contentious management practices of beef cattle production systems, surgical castration without anaesthesia and cattle’s lack of access to pasture for livestock in confined systems. This study also aimed to investigate how involvement in livestock production influences opinions, and to evaluate if different types of information would affect acceptability of these management practices.

## Materials and methods

The study was carried out in the Metropolitan Region of Santiago, Chile, and consisted of two surveys with 400 participants in each study. The first one used an online self-administered questionnaire and the second one used a face to face questionnaire. The second questionnaire had four information treatments assigned randomly, and aimed to be sex balanced in each treatment group. The Research Ethics and Safety Board at Pontificia Universidad Católica de Chile approved the study (No. 160322004) and exempted the need of consent form, due to the type of questions and the anonymity of the participants. Data were collected from June to October 2016.

### Survey 1

Data were collected via an online platform (Google Drive, https://drive.google.com/drive/), sent to email lists of different organisations (e.g. universities and hospital) and circulated through social media outlets (e.g. Facebook and Twitter). Participants were given the following information: “Surgical castration without anaesthesia is a common practice performed in male beef cattle. Typically, these animals are castrated before 6 months of age but it is also commonly applied to older animals. This practice:

- reduces sexual and aggressive behaviour between animals;
- has the risk of accidents to the people carrying out the procedure;
- avoids that animals of lower breeding value leave descendants;
- induces stress and pain to the animal, which can persist for days or weeks;
- reduces management problems and accidents with farmers, given that the animals become more docile;
- has the risk of blood loss and infection in cases were castration is carried out improperly and with inadequate post-operative care”.

The first, the third and the fifth sentences were considered positive characteristics, and the second, the forth and the sixth sentences were considered negative characteristics. After reading these characteristics related to surgical castration without anaesthesia, participants were asked if they were aware that this management practice is common in beef cattle production systems (yes, or no) and to state their opinion (support, indifferent, or oppose).

Participants were also given the following information: “In beef cattle, animals can be fattened in grassland or confined in a stall (without access to pasture). The confined system:

- allows to protect the animals against adverse climatic conditions;
- offers reduced space for movements, which limits the possibility for cattle to perform natural behaviours;
- allows better supervision of the animals (from disease and injuries, etc.);
- increases the investment costs and expenses with food and labour, etc.;
- allows to slaughter more homogeneous groups, and in a shorter period of time;
- increases the risk of disease transmission among animals”.

The first, the third and the fifth sentences were considered positive characteristics, and the second, the forth and the sixth sentences were considered negative characteristics. Similarly, after reading these characteristics related to confined system, participants were asked the same questions regarding awareness and opinion as for the other practice. The objective for giving positive and negative statements regarding both management practices was to provide a brief balance of information to all participants to ensure that all of them were answering the questionnaire from the same base knowledge.

The questionnaire also included multiple-choice demographic and socioeconomic questions covering sex, age, level of education, area of residence, source of information about animal production systems, involvement in animal production, experience in visiting a beef cattle farm, affiliation with environment and/or animal protection organisations, beef consumption frequency; and total monthly household income (participants had the option not to provide this information). To answer the later question, participants accessed a table according to the number of members living in their home, and indicated which level corresponded to the total monthly household income, which allowed classification of the socioeconomic group according to Adimark [33]: AB (upper class), C1a (accommodated medium class), C1b (emerging medium class), C2 (typical medium class), C3 (lower medium class), D (vulnerable), and E (poor).

In addition, a short knowledge-based quiz was included to evaluate the level of knowledge of participants about subjects related to beef production systems. It consisted of four true or false questions (“*In beef cattle production systems*, *the majority of calves are separated from their mothers few days after birth*”; “*The transport of animals to the slaughterhouse may affect meat quality*”; “*The diet may affect the taste of meat*”; “*Male cattle grow slower than female cattle*”). For each question, participants had to answer true, false, or do not know. All participants received an identical questionnaire, with the same order of questions and characteristics of each husbandry practice.

### Survey 2

Participants were recruited by personal invitations in public places (civil registry and solicitor office, shopping malls, medical clinic waiting areas, and national car test waiting areas). The locations were chosen due to the large waiting times of people in those places. People that were at least 18 years old and were Chilean citizens were asked to voluntarily participate in the survey answering a 4-page printed questionnaire in Spanish covering management practices in beef production systems. The researcher remained visible to answer questions but did not provide information regarding the management practices. All participants were recruited by the same person.

The questionnaire of Survey 2 was adapted from Survey 1. Participants were randomized into four treatment groups of 100 participants each. Participants received one of four types of information regarding surgical castration without anaesthesia (no information; negative; negative and positive; positive). Afterwards, they were asked if they were aware that this practice is common in beef cattle production systems (yes or no) and to state their opinion about it (support, indifferent, or opposed). *Group no information* participants were not provided with positive or negative characteristics; *Group negative* participants were provided with the three negative characteristics; *Group negative-positive* participants were provided with the three positive and the three negative characteristics; and *Group positive* participants were provided with the three positive characteristics. Within treatment groups, the order of positive and negative characteristics was randomized. Positive and negative characteristics were the same as Survey 1. All other questions and the sequence of questions remained identical to Survey 1. The same methodology was applied to the positive and negative characteristics related to the lack of access to pasture for livestock, where participants received the same type of information (no information; negative; negative-positive; positive) for the two management practices.

### Statistical analysis

In Survey 1 all data from the Google Drive platform were automatically exported to a Microsoft Excel (version 2013) sheet. Of the total participants that answered the questionnaire (n = 494), 94 were excluded from the dataset because the area of residence was not the Metropolitan Region of Santiago. In Survey 2 (n = 100/treatment), the researcher transcribed the information filled out in the questionnaires to an adapted version of the Google Drive platform used in Survey 1, which were also automatically transcribed to a Microsoft Excel (version 2013) sheet.

Descriptive statistics for the responses from Survey 1 and 2 were calculated using Microsoft Excel for Windows and all other statistical analyses were conducted using SAS 9.3. In the short knowledge-based quiz, the score was assigned as 1/-1 if the answer was correct/incorrect, respectively. If the answer was “do not know”, the score assigned was 0. The level of knowledge was calculated based on the sum of the four question results, which ranged from -4 to 4. Socioeconomic groups D (vulnerable) and E (poor) were grouped due to the low number of participants in these categories.

Spearman’s correlation coefficient was calculated to analyse the degree of association between awareness of participants regarding both management practices. Similarly, Spearman’s correlation coefficient was calculated to analyse the degree of association between opinions of participants regarding both management practices. Multinomial Logistic Regression Models were used to analyse associations between awareness, demographic/socioeconomic data and the opinion of participants towards surgical castration without anaesthesia and towards lack of access to pasture for livestock in confined systems. Opinions were considered as dependent variables. Univariate models were built to separately assess the influence of each predictor variable on the dependent variables. Predictor variables with P < 0.20 [34] were used to build multivariate models. Backward selection was used to eliminate predictor variables until only those with P < 0.10 remained in the models. Results are presented as odds ratio (ODDS) and 95% confidence interval (95% CI). Statistics associations were reported when P ≤ 0.05 and tendency when 0.05 < P ≤ 0.1.

## Results

### Survey 1

Demographic and socioeconomic data are presented in Table 1. Most of the 400 participants were under 36 years old, had at least started a graduate degree, were not involved in animal production, had visited a beef production farm before the study, and were not affiliated with environmental/animal protection organisations. The main sources of information about animal production systems cited by participants were friends (55%), Internet (52.3%), and university (49%), followed by scientific journals (19.5%), general TV and radio programs (19.3%), newspaper (15.5%), animal protection organizations (13.5%), rural TV and radio programs (9%), and others (3.5%).

**Table 1:** Demographic and socioeconomic data of Survey 1 and Survey 2 participants.

	Variable*	Survey 1 (%)	Survey 2 (%)			
			No information	Negative	Negative-Positive	Positive
		(n = 400)	(n = 100)	(n = 100)	(n = 100)	(n = 100)
**Sex**						
	**Female**	52.5	43.0	48.0	45.0	51.0
	**Male**	47.5	57.0	52.0	55.0	49.0
**Age (yr)**						
	**18–25**	36.3	23.0	20.0	19.0	15.0
	**26–35**	36.5	28.0	20.0	23.0	29.0
	**36–45**	15.0	21.0	18.0	18.0	17.0
	**46–55**	8.0	16.0	26.0	18.0	21.0
	**>56**	4.3	12.0	16.0	22.0	18.0
**Level of education**						
	**Basic and high school**	6.3	42.0	36.0	40.0	32.0
	**Graduate incomplete**	24.3	14.0	18.0	13.0	17.0
	**Graduate**	47.0	35.0	37.0	32.0	40.0
	**Postgraduate**	22.5	9.0	9.0	15.0	11.0
**Involvement in animal production**						
	**No**	70.8	96.0	95.0	96.0	93.0
	**Yes**	29.3	4.0	5.0	4.0	7.0
**Visited a beef production farm**						
	**No**	34.5	73.0	78.0	71.0	75.0
	**Yes**	65.5	27.0	22.0	29.0	25.0
**Affiliation with environment/animal protection organisations**						
	**No**	89.8	91.0	91.0	95.0	97.0
	**Yes**	10.3	9.0	9.0	5.0	3.0
**Beef consumption frequency**						
	**Never**	10.0	5.0	6.0	5.0	8.0
	**Occasionally**	18.3	25.0	23.0	21.0	28.0
	**1 to 2 times/week**	43.0	48.0	45.0	44.0	44.0
	**3 or more times/week**	28.7	22.0	26.0	30.0	20.0

* Descriptive data of “Score achieved in the short knowledge-based quiz”, and “Socioeconomic group” are not shown.

### Awareness and opinion towards both management practices

The majority of participants answered that they were aware that surgical castration without anaesthesia (79.0%) and lack of access to pasture for livestock in confined systems (83.0%) are common management practices in beef production systems in Chile. The awareness of participants regarding the two management practices were positively correlated (*r* = 0.354; P ≤ 0.0001).

The majority of participants were opposed (62.5%) to surgical castration without anaesthesia, while 20.5% were indifferent and 17% supported the practice. Similarly, the majority of participants were opposed (59.3%) to lack of access to pasture for livestock in confined systems, while 20.5% were indifferent and 20.2% supported this management practice. The opinion of participants regarding both management practices was positively correlated (*r* = 0.185; P ≤ 0.001).

### Influence of awareness regarding the practices and demographic/socioeconomic characteristics on opinion of management practices

Participants that were previously aware of surgical castration without anaesthesia, male participants, those involved in animal production, those that had visited a beef production farm and those that eat beef 3 or more times a week had higher odds of supporting versus opposing surgical castration without anaesthesia. Similarly, participants that were previously aware of surgical castration without anaesthesia, male participants, those that had visited a beef production farm and those that eat beef had higher odds of being indifferent versus opposing surgical castration without anaesthesia (P ≤ 0.05; Table 2).

**Table 2:** The number and the ratio of participants that supported or were indifferent to surgical castration without anaesthesia in Survey 1. Odds ratio (ODDS) and 95% confidence interval (95% CI) for multinomial logistic regression models of the opinion towards surgical castration without anaesthesia with the awareness and the demographic data.

			Support					Indifferent				
		Total (n = 400)	n	Ratio	ODDS	95% CI		n	Ratio	ODDS	95% CI	
**Awareness of surgical castration without anaesthesia**												
	**No**	84	2	0,028				10	0,139			
** **	**Yes**	316	66	0,371	5.344*	1.156	24.701	72	0,404	2.193*	1.004	4.789
**Sex**												
** **	**Female**	210	18	0,114				34	0,215			
** **	**Male**	190	50	0,543	2.852*	1.455	5.593	48	0,522	1.951*	1.131	3.366
**Involvement in animal production**												
** **	**No**	283	22	0,110				61	0,305			
** **	**Yes**	117	46	0,920	3.942*	2.025	7.677	21	0,420	0.812	0.430	1.533
**Visit beef production farm**												
** **	**No**	138	5					19				
** **	**Yes**	262	63		3.869*	1.352	11.072	63		2.326*	1.222	4.426
**Consumption frequency of beef**												
	**Never**	40	1	0,028				3	0,083			
** **	**Occasionally**	73	6	0,122	4.116	0.436	38.824	18	0,367	4.673*	1.252	17.440
	**1 to 2 times/week**	172	32	0,308	7.516	0.916	61.642	36	0,346	4.029*	1.137	14.275
	**3 or more times/week**	115	29	0,475	12.619*	1.505	105.828	25	0,410	4.965*	1.331	18.520

* Significantly different from reference category; *P* ≤ 0.05.

Participants that were previously aware of the lack of access to pasture for livestock in confined systems had higher odds than those that were not aware of supporting versus opposing such practice. Those involved in animal production had higher odds than those not involved of supporting or being indifferent versus opposing the lack of access to pasture for livestock in confined systems. In contrast, participants that were affiliated with environment/animal protection organisations had higher odds than those not affiliated of supporting versus opposing the practice (P ≤ 0.05; Table 3).

**Table 3:** The number and the ratio of participants that supported or were indifferent to the lack of access to pasture for livestock in confined systems in Survey 1. Odds ratio (ODDS) and 95% confidence interval (95% CI) for multimomial logistic regression models of the opinion towards lack of access to pasture for livestock in confined systems with the awareness and the demographic data.

			Support (1)					Indifferent (5)				
		Total (n = 400)	n	Ratio	ODDS	95% CI		n	Ratio	ODDS	95% CI	
**Awareness of surgical castration without anaesthesia**												
	**No**	68	3	0.059				14	0.275			
** **	**Yes**	332	78	0.419	6.282*	1.855	21.279	68	0.366	1.344	0.687	2.630
**Involvement in animal production**												
** **	**No**	283	35	0.182				56	0.292			
** **	**Yes**	117	46	1.022	5.130*	2.911	9.041	26	0.578	1.934*	1.078	3.470
**Affiliation with environment/animal protection organisations**												
** **	**No**	359	79	0.395				80	0.400			
** **	**Yes**	41	2	0.054	0.192*	0.064	0.575	2	0.054	0.097*	0.023	0.409

* Significantly different from reference category; *P* ≤ 0.05.

No associations were found between participants that supported either management practices and the other demographic data and socioeconomic groups included in the survey (P > 0.1).

Involvement in animal production was associated with awareness of surgical castration without anaesthesia (*r* = 0.248; P ≤ 0.05), awareness of the lack of access to pasture for livestock in confined systems (*r* = 0.177; P ≤ 0.05), and with having visited a beef production farm (*r* = 0.396; P ≤ 0.05).

### Survey 2

Demographic and socioeconomic data from Survey 2 are also presented in Table 1. Most participants were under 36 years old, were not involved in animal production, had not visited a beef production farm before the study and were not affiliated with environmental/animal protection organisations. The main sources of information about animal production systems were internet (51.5% of participants), general TV and radio programs (43.5%), and friends (31.5%), followed by rural TV and radio programs (14.8%), newspaper (13.3%), animal protection organizations (8.5%), university (5.5%), scientific journals (5.3%), and others (4.0%).

### Awareness and opinion towards both management practices

The majority of participants were aware that surgical castration without anaesthesia (58.8%) and lack of access to pasture for livestock (63.3%) are common management practices in beef production systems. Being aware of the two management practices was positively correlated (r = 0.341; P ≤ 0.05).

The majority of participants were opposed (79.5%) to surgical castration without anaesthesia, while 15.0% were indifferent and 5.5% supported such practice. Similarly, the majority of participants (74.8%) were opposed to lack of access to pasture for livestock in confined systems, while 17.7% were indifferent and 7.5% supported this management practice. In addition, the opinion of participants towards both management practices was positively correlated (*r* = 0.344; P ≤ 0.05).

### Influence of awareness regarding the practices and demographic/socioeconomic characteristics on opinion of management practices

Participants that were previously aware of surgical castration without anaesthesia had higher odds than those that were not aware of supporting or being indifferent versus opposing such practice (P ≤ 0.05; Table 4). No associations were found between participants that supported or were indifferent to surgical castration without anaesthesia and the other demographic data and socioeconomic groups (P > 0.1). Awareness and demographic/socioeconomic characteristics were not associated with participants supporting the lack of access to pasture for livestock in confined systems (P > 0.1).

**Table 4:** The number and the ratio of participants that support or are indifferent to surgical castration without anaesthesia in Survey 2. Odds ratio (ODDS) and 95% confidence interval (95% CI) for multimomial logistic regression models of the opinion towards surgical castration without anaesthesia with the awareness and the demographic data.

			Support (1)					Indifferent (5)				
		Total (n = 400)	n	Ratio	ODDS	95% CI		n	Ratio	ODDS	95% CI	
**Awareness of surgical castration without anaesthesia**												
	**No**	165	1	0.007				18	0.123			
	**Yes**	235	21	0.122	17.825*	2.369	134.122	42	0.244	1.981	1.093	3.589

* Significantly different from reference category; *P* ≤ 0.05.

Involvement in animal production was associated with awareness of surgical castration without anaesthesia (*r* = 0.122; P ≤ 0.05), with awareness of lack of access to pasture for livestock in confined systems (*r* = 0.132; P ≤ 0.05), and with having visited a beef production farm (*r* = 0.222; P ≤ 0.05).

### Influence of type of information on support for both management practices

The type of information (no information; negative; negative and positive; positive) received by participants did not affect their opinion regarding surgical castration without anaesthesia and the lack of access to pasture for livestock in confined systems (P > 0.1).

## Discussion

### Citizens’ opinion regarding the management practices of castration without anaesthesia and lack of access to pasture

The majority of participants from the two surveys were opposed to surgical castration without anaesthesia and lack of access to pasture for livestock in confined systems, which is in accordance with previous findings in developed and developing countries showing public concerns regarding these practices in the same and other species [5, 12, 14, 20, 35]. Reasons to support or oppose a particular farming practice may be science-based, practical, ethical or economic [36]. Citizens’ opinions are greatly influenced by perceptions of risk and ethical assessments, especially those related to human health and animal welfare [37, 38]. Other studies have identified animal welfare as a main reason behind low acceptability of animal housing and management practices [5, 22, 35], and that naturalness and humane treatment are central to what is considered good welfare [21].

To our knowledge, citizens’ opinion regarding surgical castration without anaesthesia in beef cattle has not been investigated. In the case of piglet castration, citizens preferred immunocastration, raising entire male pigs, and surgical castration with anaesthesia over surgical castration without anaesthesia, and these strategies were considered more animal-friendly alternatives [12]. Citizens believe that animals are sentient beings with capacity to suffer and have positive emotional states [5], and that imposing pain to animals is unacceptable [39]. Despite the availability of effective pain control methods, it is still common that farmers do not provide cattle with pain mitigation for surgical castration. In Chile, male beef cattle are often castrated when they achieve 250 kg or after weaning [11]. In recent years, the use of a heavy elastic band around the neck of the scrotum with both testes inside of young calves is also gaining space among farmers [11]. It is clear that both castration procedures cause pain [9, 40] but providing pain relief may involve some extra labour and expense for supplies, which is viewed as a barrier. Stakeholders that are opposed to providing pain control for dehorning and disbudding in cattle also argue that the pain experience is minimal and short lasting, and that pain control methods had little effect [29, 39]. In Canada, only 7% of beef calves younger than 6 months of age and 20% of beef calves older than 6 months of age receive anaesthesia when castrated, but these rates are still higher when the same procedure is performed by the farmer than veterinarians [41]. In the United States, veterinarians subjectively estimated castration of dairy calves younger than 6 months old as causing the least pain [42]. All these results show a disconnection between recognition of animal pain and actual use of pain control for some farm management practices known to inflict pain.

Opposition rates towards lack of access to pasture for cattle among lay citizens in other surveys [5, 20, 35] were comparable to the present study. Access to pasture is an increasingly contentious issue in countries where total confinement systems have become common [36]. Lay citizens frequently are opposed to the lack of access to pasture for cattle [5, 20, 43] and prefer systems they perceive as natural [21], where the cattle have the ability to breath fresh air, and have their health status improved [20]. Natural living tends to figure strongly in what people believe is necessary for farm animals to live a good life [35, 43]. The preference for systems where the animals are free to move and that allow animals to perform their natural behaviours [20, 21, 43–45] also seem to be a relevant concern for citizens. All this suggests a strong connection between naturalness and the concept of animal welfare [46]. With over 3 million heads of cattle, beef production in Chile is concentrated in the centre-south parts of the country [18] and the majority have access to pasture. The findings from the present study show that citizens expect beef production systems to provide access to pasture; thus, maintaining this aspect may contribute to a positive perception of farming systems and citizens’ support. Grazing is viewed by consumers as producing healthier products [20] and the rejection for zero-grazing systems is also related with concerns that the production practices may influence product quality and, potentially, human health [5].

### Influence of involvement in livestock production on opinion of management practices

In Survey 1, involvement in animal production was associated with increased acceptance of both management practices and with more participants being indifferent towards the lack of access to pasture for livestock; these respondents were mainly students of Agricultural Science or Veterinary courses, small scale producers or had professional occupations related to agriculture. Survey 2 of our study included only few participants involved in animal production (5%), which could explain the lack of association between this category and acceptance of both management practices in this survey. Stakeholders who work within the livestock industries are more accepting of contentious practices and less concerned about animal welfare than people unaffiliated with these industries [19, 47, 48], which could explain the different viewpoints of our participants. Citizens evaluate farm animal welfare as more negative than farmers, especially regarding aspects related to natural behaviour, pain, stress and availability of space [47–49]. Discussing the case of the dairy industry, Weary and von Keyserlingk [50] concluded that there is a need for sustained engagement among all industry stakeholders and the general public. This would include listening to the concerns and make changes to accommodate public expectations, a conclusion which is relevant for all animal industries.

### Influence of awareness regarding the practices and demographic/socioeconomic characteristics on opinion of management practices

Despite the study being focused on an urban population (from the Metropolitan Region of Santiago, Chile), the majority of participants in Survey 1 and 2 were aware that surgical castration without anaesthesia and lack of access to pasture for livestock in confined systems are common management practices in the beef industry. In addition, over half of participants achieved medium or high scores on the short knowledge-based quiz questions about beef production systems. This finding corroborates another Chilean survey showing that approximately 60% of people surveyed were familiar with livestock management practices [6]. Similar to Ventura et al. [35], we did not measure confidence in answers of the knowledge-based quiz but we included an option “I do not know” aiming to avoid participant guessing. In contrast, several studies reported low awareness towards animal production systems and animal welfare among citizens in other countries of Latin America [2, 5, 51–53]. In Survey 1, awareness resulted in greater acceptance and greater number of participants being indifferent towards castration without anaesthesia and greater acceptance of the lack of access to pasture for livestock; in Survey 2, awareness resulted in greater acceptance and greater number of participants being indifferent towards surgical castration without anaesthesia. Both situations contrasts with the hypothesis that public rejection of some production practices is associated with low awareness and knowledge of how food producing animals are reared [21]. This suggests that knowledge is not the sole determinant in people’s attitudes towards a particular issue [36, 38].

Participants that had visited a beef production farm before the study were more likely to support or to be indifferent towards castration without anaesthesia in Survey 1, which reinforces the different views between those who have experience and those who have no experience with farm animal production practices [36]. However, our finding contrasts with previous studies showing that people who have already visited a livestock farm were most likely to show concerns towards the welfare of those animals that are the source of food products [54]. After a farm visit, there was a decline in citizens’ attitudes toward animal welfare in dairy farms, suggesting that exposure to livestock farming may resolve certain concerns but others will persist [35]. In another Chilean survey, the majority of participants who considered animal welfare to have high level in Chile had not visited a beef production farm with a feed-lot system [6]. Therefore, it is probable that our finding is due to the association between involvement in animal production and a visit to a beef production farm. It is important to highlight that data from Survey 1 were collected via an online platform and only participants living in the Metropolitan Region of Santiago were included in the analysis. The fact that most participants had visited a beef production farm before the study could suggest that their region of origin was another than Metropolitan Region of Santiago; however, this was not asked in the surveys.

In Survey 1, but not in Survey 2, men were more likely to support castration without anaesthesia than women, which contrasts with the result from Schnettler et al. [6] who reported that Chilean men had more negative perception of livestock production practices. Usually, women have greater empathy towards animals [55], are generally more concerned about animal welfare [56], and have animal welfare as a stronger motivator for purchase [21]. Furthermore, women are generally more concerned, have more negative views towards modern farming [21], and have a stronger preference for more traditional farms [57]. All this suggests that women are more motivated by welfare concerns, consider the issue more emotively, and seem to anthropomorphise animals more than men [21]. As expected, participants that ate beef more often were also more likely to support both contentious management practices in our study, which is in line with a previous study carried out in Chile [6]. In contrast, participants affiliated with environmental/animal protection organization were less likely to support lack of access to pasture for livestock.

Belonging to any socioeconomic group had no impact on participants’ opinion on both management practices. This result contrasts with Schnettler et al. [58], who reported that Chilean citizens from middle class socioeconomic groups were more concerned about animal handling prior to slaughter. There has been a sharp reduction in the poverty rate in Chile in the last decades, decreasing from a 40% to a 14% between 1990 and 2009, which means that the medium-low class is likely to increase [59]. Chile has been one of Latin America’s fastest-growing economies over the past decade, with an economic expansion and the reduction of unemployment rate favouring this forecast for the next few years [60]. However, even with the dynamic change in socioeconomic conditions, we can expect that the overall opinion will be the same among socioeconomic groups in Chile, since it was not a factor that affected participants’ opinion.

On the other hand, higher socioeconomic status groups are more likely to consume free-range and organic meat on a frequent basis [61] and higher income is linked to an higher willingness to pay for welfare-friendly product (see review by Clark [21]). Therefore, we could expect an increase in interest for free-range and organic meat and willingness to pay for welfare-friendly products among Chilean consumers (see review by Clark [21]).

### Influence of type of information on support for both management practices

In Survey 1, all participants received the same information texts, which provided arguments for and against castration without anaesthesia and lack of access to pasture. Similar to Schuppli et al. [20] and Ventura et al. [35], the information provided to all participants was based in technical justifications employed by specialists working within the beef production industry and some common criticisms made by lay citizens. We consider that the three positive and three negative characteristics listed in the present study were designed to minimize any influence on participant perceptions toward both management practices. In Survey 2, participants received one of four types of information (no information; negative; negative-positive; positive) regarding castration without anaesthesia and lack of access to pasture. Providing negative or positive information did not result in more people opposing or supporting these practices, which contrasts with previous works that have indicated that the provision of information may influence lay citizens’ views towards management practices [5] or even consumers’ sensory perceptions [62]. In Hötzel et al. [5] survey, participants provided with information justifying the practices and discussing limitations showed higher rejection of zero-grazing systems. In contrast, UK egg consumers that received information regarding injurious pecking in free-range chicken maintained more positive attitudes towards free-range eggs than cage systems [22]. We suggest that further studies are needed to evaluate the effect of different types of information regarding these management practices in a larger population.

### Some comments on the survey methodology and other findings

It is important to highlight that these findings do not represent the views of the Chilean society, as it was based on a convenience sample of participants from the Metropolitan Region of Santiago (Chile) and participants were arguably more urban, a greater proportion were well-educated and of higher socioeconomic status than the average of Chilean population [63]. Because the current findings cannot be generalized, we urge caution in interpreting demographic associations of both surveys; however, the present study contributes novel understanding of Chilean citizens towards management practices in beef production systems, and certainly expands on existing results that where base on a more limited samples [6]. The relationships found between awareness, knowledge, demographic characteristic of respondents and support of potentially contentious farm animal practices are in general strong, and should be explored in other studies and with other examples. Furthermore, it is important to consider that, while Survey 2 recruited participants by personal invitations, Survey 1 was circulated by Internet, sent to email lists and through social media outlets, and all data were collected via an online platform, which explain that the majority had high socioeconomic status. This also explains the variation in demographic and socioeconomic group between the two surveys and the lack of effect of some aspects on Survey 2 compared to Survey 1.

## Conclusion

The majority of participants were aware that castration without anaesthesia and lack of access to pasture for livestock are common practices in beef industry, and were opposed to both management practices. Opposition was lower among participants involved in livestock production than among lay citizens. Provision of positive or negative technical assertions given by specialists working within the beef production industry and some common criticisms made by lay citizens did not influence opinions. The results show a disconnection between the views of participants recruited for this study and beef production systems that do not provide pain control for male cattle surgical castration or provide little or no access to pasture. It reinforces the importance to understand the views of citizens affiliated and not affiliated with food producing animals aiming to better harmonize beef industry practices with lay citizen expectation.
